# Aberrant choroid plexus formation in human cerebral organoids exposed to radiation

**DOI:** 10.21203/rs.3.rs-3445801/v1

**Published:** 2023-10-17

**Authors:** Marco Durante, Tamara Bender, Esther Schickel, Margot Mayer, Jürgen Debus, David Grosshans, Insa Schroeder

**Affiliations:** GSI; GSI; GSI; University of Applied Sciences Aschaffenburg; University Hospital Heidelberg; University of Texas M.D. Anderson Cancer Center; GSI

## Abstract

Brain tumor patients are commonly treated with radiotherapy, but the efficacy of the treatment is limited by its toxicity, particularly the risk of radionecrosis. We used human cerebral organoids to investigate the mechanisms and nature of postirradiation brain image changes commonly linked to necrosis. Irradiation of cerebral organoids lead to increased formation of ZO1^+^/AQP1^+^/CLN3^+^-choroid plexus (CP) structures. Increased CP formation was triggered by radiation via the NOTCH/WNT signaling pathways and associated with delayed growth and neural stem cell differentiation, but not necrosis. The effect was more pronounced in immature than in mature organoids, reflecting the clinically-observed increased radiosensitivity of the pediatric brain. Protons were more effective than X-rays at the same dose, as also observed in clinical treatments. We conclude that radiation-induced brain image-changes can be attributed to aberrant CP formation, providing a new cellular mechanism and strategy for possible countermeasures.

## Introduction

Primary brain tumors, which may arise in pediatric or adult patients are commonly treated with cranial radiation with photon or proton radiation being used. In adults, glioblastoma multiforme (GBM) is the most common primary brain tumor and is particularly challenging to treat as they exhibit a high degree of radiation and chemotherapy resistance due to their heterogeneity, stem cell-like behavior and their ability to infiltrate the normal brain^1–3^. Photon based radiotherapy is the radiation modality most used for GBM^4^. While historical studies have suggested improved disease control with dose escalated radiation therapy employing either photons or protons, these have been plagued by the development of radiation necrosis^5–8^. In pediatric patients low-grade gliomas or other tumor types are more common, and patients are often treated with high-energy protons which spare normal brain tissues to a greater extent than photons^9^. However, early studies raised concerns that pediatric brain tumor patients treated with protons experienced unexpectedly high rates of radiation necrosis^10,11^.

Radiation necrosis (RN) varies in severity and may be identified as contrast-enhancing lesions (CEL) in post-treatment magnetic resonance imaging (MRI) scans. RN is diagnosed in up to 25% of the patients treated with cranial radiation^12^. For symptomatic patients, Bevacizumab has been shown to lower the rate of RN^13^ and is recommended in several guidelines. However, the diagnosis and treatment of RN remain extremely challenging as clinically the observed CEL in brain tumor patients may represent a variety of pathophysiologies including RN, blood-brain barrier disruption (BBD) or tumor progression. Necrosis and progression occur commonly in recurrent glioblastoma and often cannot be reproducibly diagnosed by pathologists^14^. Both BBD and RN are believed to stem from radiation-induced vascular damage leading to local ischemia and hypoxia that will eventually induce necrosis or angiogenesis and subsequent brain edema due to increased permeability of the newly formed blood vessels. While BBD occurs within the first six months of radiotherapy, RN usually occurs 6–18 months after radiation exposure, but has also been observed in pediatric patients as early as 1.2 months (median time to onset,^15^) or, more commonly as a sequela of stereotactic radiotherapy, years or decades after radiotherapy^16^. BBD is believed to be transient, but can transition into RN. Progressive, untreated RN, which is characterized by large edematous lesions and pronounced clinical symptoms may be both irreversible and lethal^16^.

Contemporary clinical studies do not offer clear insights into the onset and progression of RN and to the classification of CEL as necrosis. We elected to use cerebral organoids from human pluripotent stem cells as a model to study the impact that radiation has on stem and progenitor cells within the neuronal network. As these cells mainly govern the maintenance and regeneration of the normal neuronal tissue, their contribution to the radiation-induced CEL is of great importance to avoid or treat such potential debilitating side effects. While CEL often only represent late stage RN and biopsy material is scarce and only from severe cases, cerebral organoids offer the unique opportunity to study the etiology and natural progression leading to CEL. This approach has been used with great success to uncover various neurological diseases bridging the knowledge, gained from *in vitro* and animal studies as well as human epidemiological studies^17^. Since CEL are a special concern for pediatric patients, with a high cure rate and an expected long lifespan, we used both high-energy X-rays, which are received by the majority of cancer patients, and high-energy protons, which are preferably used for treatment of pediatric malignancies.

## Results

**Figure 1:**
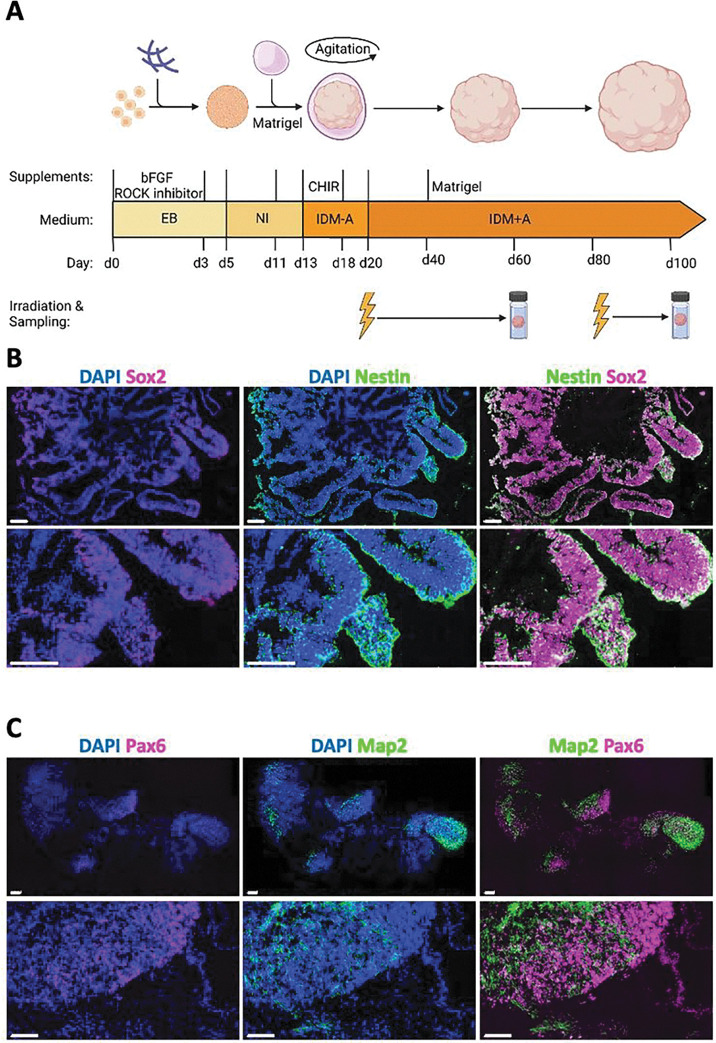
Differentiation scheme and characteristics of organoids at d20 (immature) and d80 (mature). (A) Experimental scheme (created with BioRender.com). (B) Representative immunofluorescence stainings of SOX2 (magenta) and Nestin (green) in d20 cerebral organoids, nuclei stained with DAPI, scale bar: 100 μm. (C) Representative immunofluorescence stainings of SOX2 (magenta) and Nestin (green) in d80 cerebral organoids, nuclei stained with DAPI, scale bar: 100 μm.

As CEL occur in various brain regions depending on the localization of the tumor and the radiation field, we used an unguided differentiation approach according to Lancaster et al.^18,19^ to generate cerebral organoids with a maximum diversity of initial progenitor cells capable to form a variety of regional identities of the human brain (Fig. 1A). Organoids were irradiated at day (d) 20, when they mainly comprise of sex determining region Y (SRY)-box 2 (SOX2)/Nestin-positive neural progenitors clustered in ventricular zone-like rosettes (immature organoids, Fig. 1B) with the first neurons to appear^17^. Additionally, organoids were irradiated at d80, when the neuronal network has matured and expresses the neuronal marker microtubule-associated protein 2 (MAP2) next to regions with paired box protein-6 (PAX6)-positive neuronal progenitors (mature organoids, Fig. 1C) and show electrical activity (see Suppl. Figure 1). As d20 organoids still differentiate substantially, changing their cellular composition accordingly, samples were taken at around d60. In contrast, the more mature organoids irradiated at d80 were followed up at d100.

### Exposure to radiation results in growth retardation, but is not correlated to necrosis

**Figure 2:**
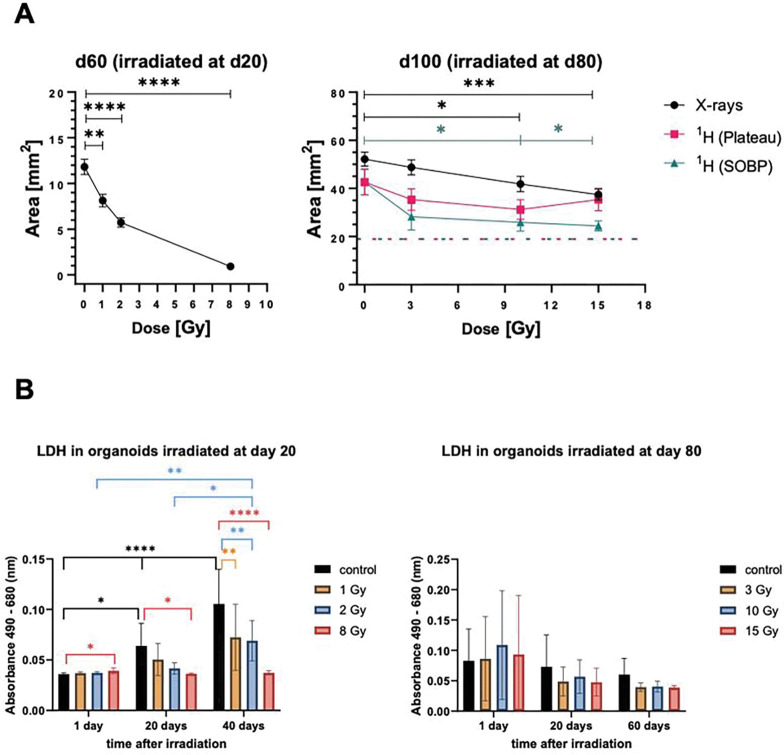
Size measurement and analysis of necrosis in organoids. (A) The organoid size measured as area [mm^2^] for organoids irradiated at d20 (immature) and at d80 (mature) measured 40 and 20 days post-irradiation (d60 and d100), respectively, using different radiation qualities and doses: X-rays: 1 – 8 Gy for d20 organoids and 3 – 15 Gy for d80 organoids; protons in SOBP or plateau, respectively: 3 – 15 Gy for d80 organoids in comparison to their respective sham-irradiated controls. Dashed lines delineate the size of the controls at the day of irradiation. Statistical analysis was done either by Brown-Forsythe and Welch ANOVA or one-way ANOVA with Dunnettś post-test. Data are presented as mean ± SEM for N = 3 and n = 25–36 (X-rays, d20), N = 4 and n = 40–54 (X-rays, d80) and N = 1 and n = 10 (protons, d80). *p<0.05, **p<0.01, ***p<0.001, ****<0.0001. (B) Lactate dehydrogenase (LDH) levels secreted by organoids irradiated with X-rays at d20 (1 – 8 Gy), measured 1, 20 and 40 days post-irradiation, and secreted by organoids irradiated at d80 (3 – 15 Gy), measured 1, 20 and 60 days post-irradiation. LDH level is directly proportional to absorbance values measured at 490 nm and subtracted from background values measured at 680 nm. Statistical analysis was done by two-way ANOVA with Tukeýs post-test. Data are presented as mean ± SD for N = 3 and n = 3. *p<0.05, **p<0.01, ****p<0.0001.

To study the impact of radiation on the size of the cerebral organoids, X-rays were used as a reference radiation. As fetal/neonatal neural stem cells are radiosensitive^20^, doses of 1–8 Gy were used. For the more mature organoids, 3–15 Gy were applied. All samples were compared to sham-irradiated controls. In addition, d80 organoids were irradiated with high-energy protons (3–15 Gy), commonly used for radiotherapy of pediatric cancers^21^. The results of the size measurements are shown in Fig. 2A. Compared to the controls at the day of irradiation (dotted lines), all sham controls showed an increase in size. Organoids irradiated at d20 displayed a significant dose-dependent decrease in size at d60 when compared to the sham-controls. In the highest dose-cohorts, the size was even lower than in the d20 samples (analyzed 40d prior). Mature organoids irradiated on d80 responded less drastic. Here significant growth retardation was only observed in samples subjected to 10 or 15 Gy X-rays or proton irradiation in the Spread Out Bragg Peak (SOBP), corresponding to the irradiation of the tumor bed, while irradiation in the entrance (plateau, corresponding to the surrounding normal tissue) was not significantly altered compared to the sham controls. Measuring necrosis via lactate dehydrogenase (LDH) release in samples irradiated with X-rays at d20 revealed highest rates of necrosis in the sham controls, but significantly lower levels in the irradiated samples 20 and 40d after exposure. In samples irradiated at d80, no significant changes could be observed (Fig. 2B).

### Irradiation leads to formation of liquid-filled cavities

**Figure 3:**
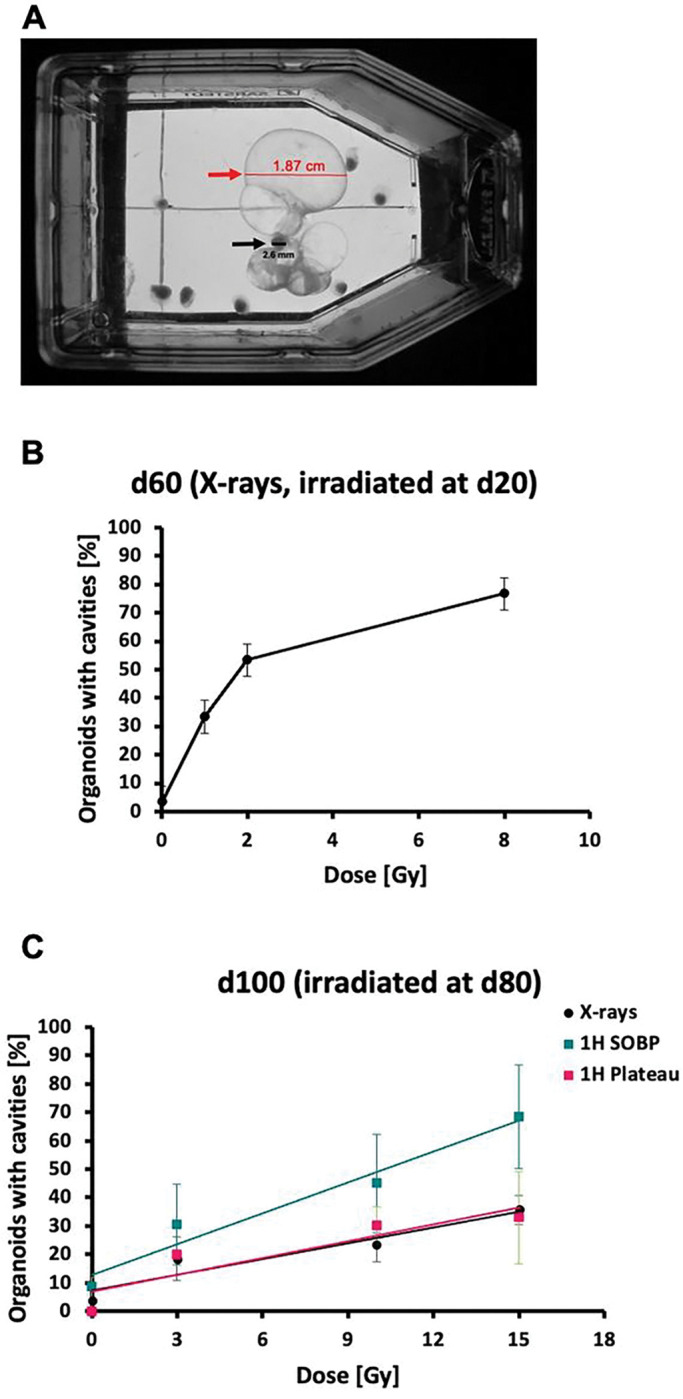
Cavity formation in cerebral organoids after irradiation with various radiation qualities. (A) Organoid, irradiated at d80 with 15 Gy protons (Plateau phase), displays massive liquid filled cavities. (B) Percentage of organoids displaying cavities after irradiation with X-rays at d20 (1 – 8 Gy) and (C) with various radiation qualities: X-rays, protons in SOBP and in plateau, respectively at d80 (3 – 15 Gy). Quantitative analysis of the dose-response curve and relative biological effectiveness are reported in Supplementary Table 1. Data are presented as mean ± SD, N = 2–3, n = 10.

Irradiation of organoids led to the formation of liquid-filled cavities that in general had a diameter of between 100–800 μm, but could become as large as 1.87 cm in diameter with a diameter of the corresponding organoid of 2.6 mm only (Fig. 3A). In accordance with the growth retardation, occurrence of these cavities (Fig. 3B) was highest in the samples irradiated at d20. In the samples irradiated at d80 (Fig. 3C), protons in the SOBP were more effective than X-rays and protons in the plateau phase at the same dose. The slope of the dose-response curve and the relative biological effectiveness of proton beams compared to X-rays are reported in the Supplementary Table 1.

### Radiation-induced liquid-filled cavities represent choroid plexus

**Figure 4:**
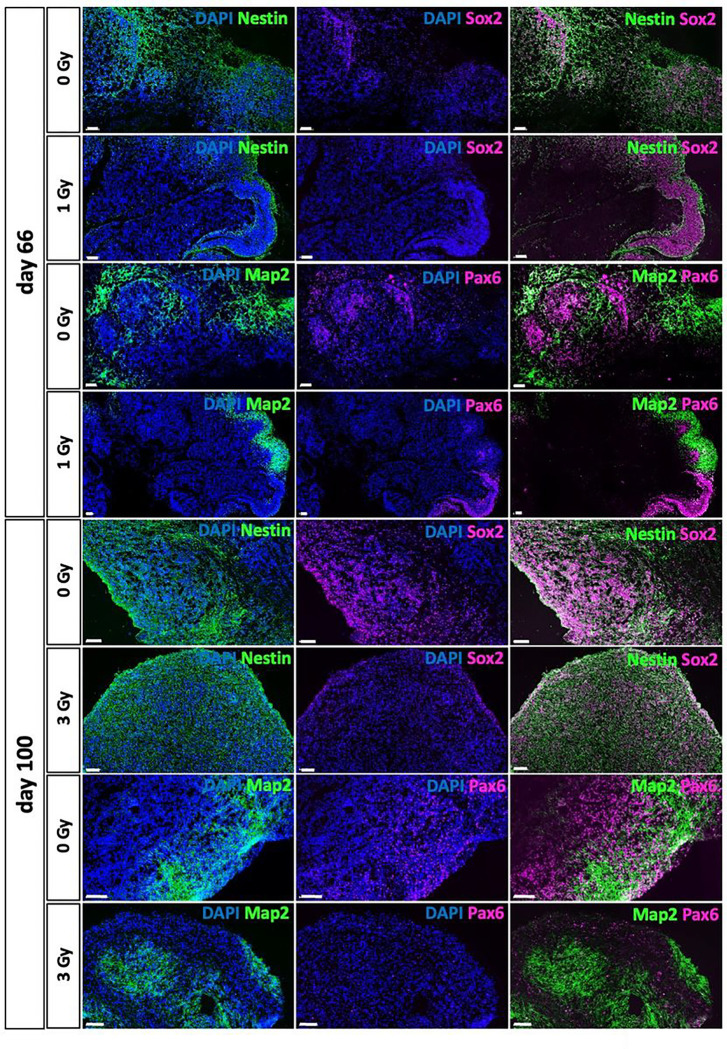
Protein expression of markers for neural stem and progenitor cells and mature neurons in irradiated organoids and their sham controls. Representative immunofluorescence stainings of SOX2 (magenta) and Nestin (green) or PAX6 (magenta) and MAP2 (green) at d66 (irradiation at d20) or d100 (irradiation at d80). Nuclei stained with DAPI, scale bar: 100 μm.

**Figure 5:**
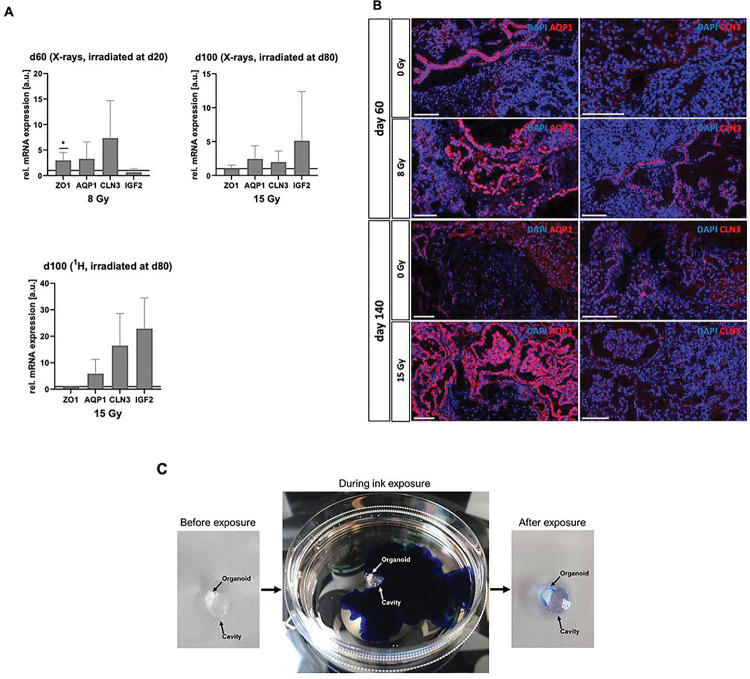
Choroid plexus (CP) formation in irradiated cerebral organoids. (A) Relative mRNA expression of the CP markers ZO1, AQP1, CLN3 and IGF2 in d66 organoids subjected to 8 Gy X-rays at d20, mean ± SEM, N = 4, n = 3 and in d100 organoids subjected to either 15 Gy X-rays or 15 Gy protons at d80 (mean ± SEM, N = 3 – 4, n = 3 for X-rays and N = 2 – 3, n = 3 for protons). Statistical analysis was done by two-tailed Welchś t-test, * p<0.05. The lines indicate the levels in the sham-irradiated controls. (B) Representative immunofluorescence stainings of the CP markers AQP1 and CLN3 (red), scale bar = 100 μm, (C) Cerebral organoid after ink-based barrier test.

The choroid plexus comprises of highly fenestrated epithelial tissue that forms the blood-cerebrospinal fluid (CSF) barrier and is the production site for the CSF in the vertebrate brain^22^. It regulates the entry of compounds into the brain, its development and function. As the CP epithelium arises from multipotent neuroepithelial stem cells and CP in turn also supports these stem cells^22^, we examined the distribution of neuroepithelial stem cells in organoids before and after irradiation. Non-irradiated controls at d66 showed progenitor cells positive for SOX2, Nestin or PAX6 intermingled with neuronal cells partially positive for MAP2, the marker of mature neurons. In irradiated organoids those stem cells were still clustered in ventricular zone-like rosettes indicating an earlier differentiation stage/more immature differentiation status as would be found at d20 (Fig. 1B). Figure 4 shows these differences exemplarily for an organoid at d66 irradiated with 1Gy X-rays compared to the sham-irradiated control. In organoids at d100, which were irradiated at d80, these proliferation zones were not identified, although some cells still showed weak expression of SOX2 and higher expression of Nestin (Fig. 4). As apparently irradiated organoids retained neuroepithelial stem cells in rosettes that could still form CP and the observed cavities showed similarities to CP structures, we analyzed common choroid plexus markers zona occludens (ZO) 1, aquaporin (AQP) 1, claudin (CLDN) 3 as well as insulin-like growth factor (IGF) 2, a marker which is highly enriched during brain development^23^ and is restricted to dark cells, a subtype of the CP epithelium, which has been uncovered using CP organoids^24^. Compared to sham-controls, the levels of ZO1, AQP1 and CLN3 mRNA were upregulated in samples subjected to 8 Gy X-rays (Fig. 5A) while IGF2 was slightly less expressed. In d80 irradiated organoids (Fig. 5A), AQP1, CLN3 and IGF2 mRNA levels were elevated after irradiation with 15 Gy X-rays or protons. ZO1 was equally distributed in sham controls and irradiated samples. The existence of CP structures with their distinct morphology as a monolayer of epithelial cells in organoids subjected to irradiation was confirmed in immunofluorescence staining against AQP1 and CLDN3, which are only found in CP (Fig. 5B). These structures were also positive for the gap junction protein ZO1. However, whereas the fluorescence signal for CLN3 was restricted to CP structures, ZO1 was found in both, ventricular/neuroepithelial proliferation zones and in CP (Figure S2). The barrier-function of the cell-layer lining the cavities was directly assessed by examining the entry of ink from the medium into the cavities. However, the ink was completely excluded from the cavities, proving intact barrier characteristics over the observed time of up to 5 min (Fig. 5C).

### Altered NOTCH and WNT signaling reflects organoids response to irradiation and CP formation

**Figure 6:**
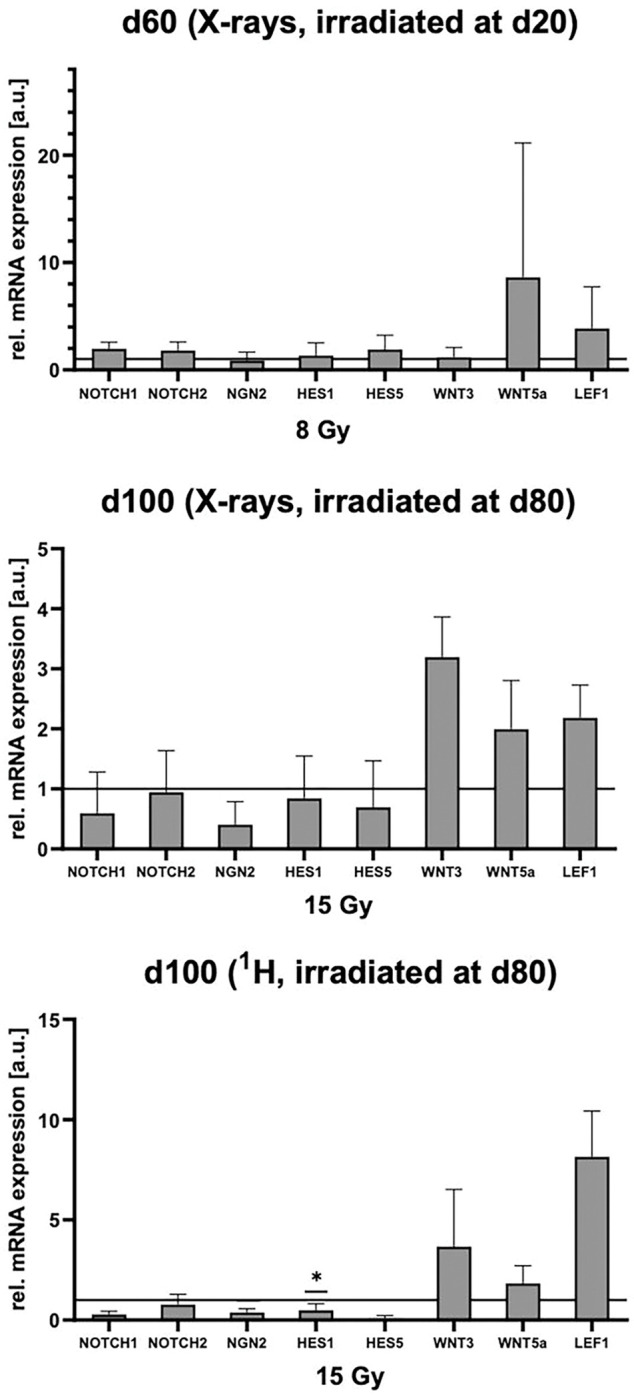
Notch/Hes and WNT signaling in irradiated cerebral organoids. (A) Relative mRNA expression of the markers NOTCH1, NOTCH2, NGN2, HES1, HES5, WNT3, WNT5a and LEF1 in d66 organoids subjected to 8 Gy X-rays at d20, mean ± SEM, N = 4, n = 3. (B) Relative mRNA expression of the markers NOTCH1, NOTCH2, NGN2, HES1 HES5, WNT3, WNT5a and LEF1 in d100 organoids subjected to 15 Gy X-rays, mean ± SEM, N = 4, n = 3. (C) Relative mRNA expression of the markers NOTCH1, NOTCH2, NGN2, HES1 ES5, WNT3, WNT5a and LEF1 in d100 organoids subjected to 15 Gy protons (SOBP) at d80, mean ± SD, N = 1, n = 3. Statistical analysis was done by two-tailed Welchś t-test, * p<0.05. The lines indicate the levels in the sham controls.

CP formation from neuroepithelial cells and its underlying mechanisms are unclear. However, the role of neurogenic locus notch homolog protein (NOTCH) signaling has been established as NOTCH directly induces hairy and enhancer of split (HES) 1, 3 and 5, whose expression leads to the specification of CP epithelium^22^ at the expense of neurogenin (NGN) 2. Thus, we assessed the mRNA expression of NOTCH1 and 2, NGN2 as well as HES1 and 5 (Fig. 6). NOTCH1 and 2, and HES1 and 5 were slightly increased in organoids irradiated at d20. In organoids exposed to X-ray or proton irradiation (SOBP) at d80, the expression of NOTCH1 and 2, HES1 and HES5 were lower than in the sham controls. This agrees with a generally weaker reaction to the irradiation requiring considerably higher doses compared to the organoids irradiated at d20. NGN2 was equally distributed in sham controls and samples irradiated with X-rays at d20, while it was decreased in samples irradiated with X-rays and protons at d80, respectively.

WNT signaling is known to also play a role in CP formation. WNT3a null mutants exhibit smaller CP areas, while treatment with WNT3 supports BMP4 mediated CP derivation from embryonic stem cells^25^. WNT5a promotes the formation of the tight epithelium in the developing CP and lack of WNT5a is associated with reduced CP size and complexity and loss of apicobasally polarized morphology^26^. As WNT signaling via b-catenin acts as a co-activator of lymphoid enhancer factor 1(LEF1)/TCF transcription factors, we included LEF1 in the analyses^27^. mRNA level of WNT5a and LEF1 were elevated in both, organoids irradiated at d20 and d80 irrespective of the radiation quality. While mRNA level of WNT3 were elevated in organoids irradiated at d80, this increase was not observed in organoids irradiated at d20 (Fig. 6).

## Discussion

Cerebral organoids have been used to mimic the onset and progression of a number of neurological diseases^17^ including the dysregulation of brain and CP cell types in patients with severe SARS-CoV-2 infections^28,29^. Here, we use cerebral organoids to elucidate the nature of contrast-enhancing lesions (CEL) that are generally attributed to radiation necrosis as a result of damage to the vasculature of the brain and the blood brain barrier. Using cerebral organoids generated via unguided differentiation, we show here that CEL appearance involves the blood-cerebrospinal fluid barrier or choroid plexus. The fact that necrosis in the irradiated samples is lower than in the non-irradiated organoids stems from an intrinsic issue of the growing organoids: As they enlarge reaching diameters of several millimeter they exceed the limit for passive diffusion of oxygen and sufficient nutrient supply, resulting in necrotic cells at the core^30^. Initial radiation-induced cell killing leading to the observed growth retardation, thus, resulted in an alleviated necrosis in the irradiated organoids compared to the controls.

The formation of liquid filled cavities or cysts is clearly attributable to the radiation impact. Cerebral organoids generated in an unguided differentiation process according to the protocol by Lancaster et al.^18,19^ are capable of developing CP. However, this process is rare and in our study occurred in less than 10% of all control organoids formed compared to up to 50–70% in irradiated organoids. Indeed, to generate CP organoids, dorsalization has to be induced using bone morphogenic protein (BMP) 4^24^ that *in vivo* stimulates epithelial cell formation within the dorsal midline by repressing forkhead box (FOX) G1^31^, which supports neural stem/progenitor cell proliferation^32^. The formation of liquid filled cavities in such induced conditions is observed after differentiation for more than 28 days and follows the appearance of characteristic cuboidal epithelium as has been shown in our study as well. As such, in our study the radiation impact mimicked the guided differentiation of CP organoids as judged also by the expression of CP markers such as AQP1, the water channel essential for CSF production and the tight junction proteins ZO1 and CLN3, which ensure the integrity of the CP^31^. While ZO1, even though being a more general marker of tight junctions, is found in neuroepithelial proliferation zones and in CP, the morphology of the latter is clearly distinguishable, rendering ZO1 a suitable marker for CP in addition to the others used in this study.

The existence of members of the NOTCH/HES signaling pathway and especially the induction of WNT/BMP signaling, that are both required for the regulation of CP formation *in vivo* ^22,25,26^, together with unchanging or decreased neural NGN2 have been identified as being the cause for the altered differentiation process by the radiation impact. For the NOTCH signaling this effect was more pronounced in the immature organoids than in the mature ones, while WNT signaling induction was observed in all organoids irrespective of their maturation status and the type of irradiation used. This is in agreeance with findings that the use of the WNT activator CHIR in combination with BMB4 is sufficient to induce CP in cerebral organoids^24^

This opens up an entire new interpretation of the CEL seen in brain tumor patients that received radiation therapy, which is substantiated by two clinical studies. Recently, Eulitz et al. showed an increased radiosensitivity in the periventricular region correlating with an increase in late radiation-induced brain injuries (RIBI) when evaluating consecutive MRI scans as a follow up after proton therapy in patients with glioma^33^. Median distances of the RIBI volume centers to the cerebral ventricles, where the CP is located, and to the clinical target volume border were 2.1 mm and 1.3 mm, respectively. The same correlation of CEL formation and proximity to the periventricular region has been observed in a second study^34^. From our findings and the onset of RIBI/CEL in the ventricular region, an involvement of the CP in the onset of such lesions is likely. We propose that radiation in rare cases induces focal CP formation from neuroepithelial stem cells leading to the typical frond-like structures with increasing CSF production that have great resemblance to the CEL seen in MRI scans of patients who received cerebral radiotherapy. Even though in our short-time observations, CP barrier function was intact, arguing against a possible *in vivo* leakage of contrast-enhancing agents into the CP cavities, a comparison of 5 min and 4 h post-intravenous (IV) administration of a single dose of gadolinium-based contrast agents in a small cohort of patients with clinically suspected endolymphatic hydrops revealed significant leakage of contrast agents into the CSF spaces only at 4 h, but not at 5 min post-IV^35^. Thus, a leakage into the newly formed CP cavities could still be possible *in vitro* and *in vivo*. *In vivo*, such leakage was observed in patients with stroke, in those with previous surgery, and in those with high-grade gliomas^36^. It has been shown that embryonic as well as adult CSF can support adult neural stem cells in culture via IGF2^23^. IGF2 levels are attributed largely to CP secretion and peak during brain development. CP/CSF-IGF2 simulates cell divisions by binding to receptors on the surface of neural stem cells^23^ In our study, IGF2 was found to be upregulated in mature organoids demonstrating CSF production and secretion. Likewise, neurogenesis can occur from neural precursors within the developing choroid plexus^37^. Therefore, the formation of CP at the expense of newly formed neurons as a response of neuroepithelial stem cells to irradiation is possible. The late production of CSF may thus explain the late onset, but progressive enlargement of CEL. If such a route would be responsible for the onset of CEL, it could also explain, why pediatric patients may be more severely affected than adult patients, as the pediatric brain still exhibits a higher plasticity than the adult brain^38^. In addition, the efficacy of the current approach to treat CEL with Bevacizumab would not contradict the involvement of CP formation. Even though this humanized anti-VEGF monoclonal antibody is applied to inhibit vascular growth and to normalize the blood-brain barrier^39^, thus alleviating a brain damage caused by radiation-induced vascular injury, it would also impact CP cells as VEGF is required for CP maintenance and VEGF receptors are found in adult CP^40^. Likewise, Thalidomide has been found to restore the blood-brain barrier and cerebral perfusion in a mouse model of RIBI. This restored function was attributed to induction of platelet-derived growth factor receptor β (PDGFRβ) expression and subsequent rescue of pericytes^41^. However, Thalidomide also downregulates LEF1, the co-activator of the Wnt/β-catenin signaling required for CP formation as well as IGF2, which is crucial for CP generation and CSF secretion (see above). Therefore, like Bevazizumab, Thalidomide has a potential inhibiting effect on CP formation/progression and CSF production^42^. If focal/excessive CP/CSF formation would be the cause of at least some of the CEL observed in brain tumor patients, additional drugs such as topiramate or the diuretics acetazolamide and furosemide would be a treatment option as well, as they decrease secretion of CSF from the CP^43^.

## Conclusion

The formation of CP and underlying alterations in the NOTCH/WNT pathways in response to radiation as a cellular stressor has not been previously described. It appears irrespective of the radiation quality, but strongly depends on the age of the neuronal tissue that is exposed. CP formation offers a novel interpretation of the initiation and progression of radiation-induced CEL seen in brain cancer patients and opens up possible new treatment regimes.

## Methods

### Human embryonic stem cell culture

The feeder-independent hES cell line WA09-FI (H9) was used for all studies presented as approved according to § 4 of the German Stem Cell Act (registry numbers 3.04.02/0125 and 3.04.02/0125-E01). The line was originally generated by the group of Dr. James Thomson at the University of Wisconsin ^44^. H9 cells were obtained from the WiCell Research Institute, Wisconsin, USA, at passage 23 and were used for experiments in passages 43–53. Cells were routinely cultured on Laminin-521-coated culture dishes (BioLamina, #600962, 10 μg/ml) in mTeSR1 medium (STEMCELL Technologies) supplemented with 50 U/ml penicillin and 5 μg/ml streptomycin (Merck, #A2212) and passaged every 7d using ReleSR (STEMCELL Technologies, #05872). Briefly, medium was aspirated and cells were rinsed first with 2 ml PBS and then with 200 μl ReleSR. After aspiration of the non-enzymatic passaging reagent, cells were incubated for 2–3 min at 37°C to detach only pluripotent cells. Detachment was stopped by addition of pre-warmed mTeSR1 medium and cells were seeded to about 1.0 ×10^5^ cells per cm^2^.

### Generation of cerebral organoids

Cerebral organoids were generated as previously described ^18,19^. Briefly, for the generation of embryonic bodies (EBs), H9 cells were detached using ReLeSR (Stemcell Technologies) for 3 min at 37°C. 18000 cells in EB medium (EBM) with addition of 4 ng/ml basic fibroblast growth factor (bFGF) and 50 μM Rho-associated protein kinase (ROCK) inhibitor (Tocris Bioscience) were plated into each well of an U-bottom suspension plate (Sarstedt) pre-coated with anti-adherence solution (Stemcell Technologies) for 5 min, 1000 rpm, at RT. Medium was changed to EBM without additional factors on day 3 of culture. EBs were transferred to neural induction (NI) medium in 6-cm dish (Sarstedt) to form neuroepithelial tissues on day 5 of culture. Fresh NI medium was added every second day after transfer. EBs were embedded into Matrigel (Corning) droplets on day 11 and cultured in NI medium until day 13 when it was changed to improved differentiation medium without vitamin A (IDM-A) with addition of 3 μM CHIR99021 (Biovision). Medium was then changed every second day until day 18. EBs were then cultured on an orbital shaker in T-25 flasks. Medium was switched to improved differentiation medium with vitamin A (IDM + A) on day 20 of culture, and then changed every 3–4 days. On day 40 of culture, 20 μl/ml Matrigel was added to IDM + A medium.

### Detection of LDH release from necrotic cells

To detect extracellular lactate dehydrogenase (LDH) released from necrotic cells in the media, CyQUANT^™^ LDH Cytotoxicity Assay Kit (Invitrogen) was used according to manufacture’s instructions. Briefly, culture medium was collected at certain time points (1 day, 20 days and 40 or 60 days) after X-ray irradiation with different doses, i.e. 1 Gy, 2 Gy and 8 Gy for organoids irradiated on day 20 of culture or 3 Gy, 10 Gy and 15 Gy for organoids irradiated on day 80 of culture. On the day of assay, 50 μl of each sample medium, previously stored at −80°C, was added to a 96-well black clear-bottom plate in duplicate wells. Then, 50 μl of Reaction Mixture from the kit was added to each sample and incubated at room temperature for 30 minutes in the dark to enable conversion of lactate to pyruvate in a reaction catalyzed by LDH where NAD + is reduced to NADH. The reaction was stopped by adding 50 μl Stop Solution from the kit. The absorbance of red formazan product generated by reduction of a tetrazolium by diaphorase, which also oxidizes NADH, was measured spectrophotometrically at 490 nm. 680-nm absorbance value (background) was subtracted from the 490-nm absorbance. Obtained absorbance values are directly proportional to the amount of LDH released into the media.

### Irradiation of cerebral organoids

At d20 or d80 of differentiation, 5–10 cerebral organoids in T25 suspension flasks were subjected either to X-rays or protons in a dose range of 1–8 Gy (X-rays, d20) or 3–15 Gy (X-rays and protons). X-ray irradiation was performed using a MXR320/26 X-ray tube (250 kV, 16 mA) at the GSI Helmholtzzentrum für Schwerionenforschung. Proton beams (250 MeV initial energy) were used at the Heidelberg Ion Therapy (HIT) Center or at the GSI Helmholtzzentrum für Schwerionenforschung. Samples were exposed to protons either in a 30 mm Spread Out Bragg Peak (SOBP, LET = 2.5–8.9 keV/μm) representing the irradiation field of the tumor plus margin; or in the plateau region (LET = 0.9–1.5 keV/μm) representing the surrounding normal tissue. Controls were sham-irradiated. Medium was exchanged immediately after irradiation or up to 1h thereafter.

### Size measurements and quantification of cavity formation

For the measurement of the organoid size, pictures of 10 organoids were taken at d20 and d80 (time of irradiation) and d60 and d100 (40 and 20d post-irradiation, respectively) using a standard digital camera (Sony DSC-W220). The organoid area was measured using ImageJ.

The number of organoids with visible cavities was counted and presented as a percentage of the overall number of organoids.

### Analyses of cavity permeability

To examine the barrier function, an ink test was carried out. For this purpose, 6 drops of dark blue writing ink were carefully added to the cultivation medium under the microscope to observe the permeability of the cavity walls. Videos were recorded for 40 sec at a frame rate of 31.68 frames per second using the microscope Eclipse Ts2 microscope (Nikon). Maximum observation time was ≤ 5 min.

### Immunofluorescence

For immunofluorescence staining, organoids were fixed in 3.7% paraformaldehyde (Carl Roth) at 4°C overnight and then washed three times for 5 min with PBS. Organoids were dehydrated in sucrose (Sigma) gradient (7–60% sucrose in PBS) for 4 hours (for 7%, 10% and 40% sucrose) or overnight (for 30% and 60% sucrose). Organoids were then embedded in 7.5% gelatin (Neolab)/10% sucrose using in house 3D-printed PDMS embedding molds. Embedded organoids were frozen on dry ice and stored at −80°C prior to being cryosectioned at 10 μm using CM1860 cryostat (Leica Biosystems). Cryosections were first blocked and permeabilized in 0.5% Triton X-100 (ThermoFisher Scientific)/1% BSA (Carl Roth) in PBS for 30 min, and then blocked with 1% BSA in PBS for another 30 min at room temperature (RT). Cryosections were incubated with primary antibodies in 1% BSA in PBS for 1 h at RT or at 4°C overnight. After being washed three times for 5 min with PBS, cryosections were incubated with secondary antibodies in 1% BSA for 1h at RT followed by incubation with 5 μg/ml DAPI for 4 min for nuclei staining. Then the cryosections were washed two times for 5 min in PBS followed by two wash steps for 5min with Millipore water prior to mounting with fluorescence mounting medium (Dako). The antibodies used for immunofluorescence are listed in Supplementary Tables 2–4. Images were captured with a fluorescence microscope Zeiss Axio Imager.Z2 equipped with Metafer5 software (Metasystems). The stainings were performed on samples from three independent preparations with at least three organoids per group. Images were processed with ImageJ (v1.53i, National Institute of Health (NIH)).

### Real time RT-PCR analysis

For each condition to be analyzed 3–5 organoids were collected in QIAzol Lysis Reagent (Qiagen, #79306) and total RNA was isolated using the Qiagen RNeasy Mini Kit (#74106) according to the manufacture’s instructions including a DNA removal step using the RNase-free DNase Set (Qiagen, #79254). 2μg RNA were reverse-transcribed via the RevertAid RT Kit (Life Technologies, #K1691). Relative RNA expression was analyzed using the Hot FIREPol EvaGreen qPCR Mix Plus from Solis Biodyne (08–24-0000S) and the QunatStudio 3 Real-Time PCR System. Human fetal and adult brain mRNA served as controls, all target expression levels were normalized to 18S rRNA. Primer sequences can be found in Supplement Table 5.

### Statistics

Statistical comparisons were performed using GraphPad Prism (v 9.3.1) as stated in figure legends and included unpaired two-tailed test with or without Welch correction, Brown-Forsythe/Welch or mixed ANOVA model either with Dunnett’s or Tukeýs post-test. Samples of organoids were randomly assigned to different treatments. No statistical methods were used to pre-determine samples sizes. Because of the nature of the treatment (irradiation), data collection and analysis were not performed blind to the conditions of the experiments.

## References

[1] Ghosh D., Nandi S. & Bhattacharjee S. Combination therapy to checkmate Glioblastoma: clinical challenges and advances. Clin Transl Med 7, 33, doi:10.1186/s40169-018-0211-8 (2018). 30327965 PMC6191404

[2] Gimple R. C., Bhargava S., Dixit D. & Rich J. N. Glioblastoma stem cells: lessons from the tumor hierarchy in a lethal cancer. Genes Dev 33, 591–609, doi:10.1101/gad.324301.119 (2019). 31160393 PMC6546059

[3] Nakada M. Molecular targets of glioma invasion. Cell Mol Life Sci 64, 458–478, doi:10.1007/s00018-007-6342-5 (2007). 17260089 PMC11138430

[4] van Solinge T. S., Nieland L., Chiocca E. A. & Broekman M. L. D. Advances in local therapy for glioblastoma - taking the fight to the tumour. Nat Rev Neurol 18, 221–236, doi:10.1038/s41582-022-00621-0 (2022). 35277681 PMC10359969

[5] Fitzek M. M. Accelerated fractionated proton/photon irradiation to 90 cobalt gray equivalent for glioblastoma multiforme: results of a phase II prospective trial. J Neurosurg 91, 251–260, doi:10.3171/jns.1999.91.2.0251 (1999). 10433313

[6] Matsuda M. Prognostic factors in glioblastoma multiforme patients receiving high-dose particle radiotherapy or conventional radiotherapy. Br J Radiol 84 Spec No 1, S54–60, doi:10.1259/bjr/29022270 (2011). 21427185 PMC3473893

[7] Mizumoto M. Phase I/II trial of hyperfractionated concomitant boost proton radiotherapy for supratentorial glioblastoma multiforme. Int J Radiat Oncol Biol Phys 77, 98–105, doi:10.1016/j.ijrobp.2009.04.054 (2010). 19695794

[8] Tsien C. I. Concurrent temozolomide and dose-escalated intensity-modulated radiation therapy in newly diagnosed glioblastoma. Clin Cancer Res 18, 273–279, doi:10.1158/1078-0432.CCR-11-2073 (2012). 22065084 PMC3266840

[9] Durante M., Orecchia R. & Loeffler J. S. Charged-particle therapy in cancer: clinical uses and future perspectives. Nat Rev Clin Oncol 14, 483–495, doi:10.1038/nrclinonc.2017.30 (2017). 28290489

[10] Gunther J. R. Imaging Changes in Pediatric Intracranial Ependymoma Patients Treated With Proton Beam Radiation Therapy Compared to Intensity Modulated Radiation Therapy. Int J Radiat Oncol Biol Phys 93, 54–63, doi:10.1016/j.ijrobp.2015.05.018 (2015). 26279024

[11] Kralik S. F. Radiation Necrosis in Pediatric Patients with Brain Tumors Treated with Proton Radiotherapy. AJNR Am J Neuroradiol 36, 1572–1578, doi:10.3174/ajnr.A4333 (2015). 26138138 PMC7964686

[12] Gibson E. & Monje M. Effect of cancer therapy on neural stem cells: implications for cognitive function. Curr Opin Oncol 24, 672–678, doi:10.1097/CCO.0b013e3283571a8e (2012). 22913969 PMC10234770

[13] Kulinich D. P. Radiotherapy versus combination radiotherapy-bevacizumab for the treatment of recurrent high-grade glioma: a systematic review. Acta Neurochir (Wien) 163, 1921–1934, doi:10.1007/s00701-021-04794-3 (2021). 33796887 PMC8195900

[14] Holdhoff M. The consistency of neuropathological diagnoses in patients undergoing surgery for suspected recurrence of glioblastoma. J Neurooncol 141, 347–354, doi:10.1007/s11060-018-03037-3 (2019). 30414096 PMC6342857

[15] Plimpton S. R. Cerebral radiation necrosis in pediatric patients. Pediatr Hematol Oncol 32, 78–83, doi:10.3109/08880018.2013.791738 (2015). 23647507

[16] Bernhardt D. DEGRO practical guideline for central nervous system radiation necrosis part 1: classification and a multistep approach for diagnosis. Strahlenther Onkol 198, 873–883, doi:10.1007/s00066-022-01994-3 (2022). 36038669 PMC9515024

[17] Eichmuller O. L. & Knoblich J. A. Human cerebral organoids - a new tool for clinical neurology research. Nat Rev Neurol 18, 661–680, doi:10.1038/s41582-022-00723-9 (2022). 36253568 PMC9576133

[18] Lancaster M. A. Guided self-organization and cortical plate formation in human brain organoids. Nat Biotechnol 35, 659–666, doi:10.1038/nbt.3906 (2017). 28562594 PMC5824977

[19] Lancaster M. A. & Knoblich J. A. Generation of cerebral organoids from human pluripotent stem cells. Nat Protoc 9, 2329–2340, doi:10.1038/nprot.2014.158 (2014). 25188634 PMC4160653

[20] Michaelidesova A., Konirova J., Bartunek P. & Zikova M. Effects of Radiation Therapy on Neural Stem Cells. Genes (Basel) 10, doi:10.3390/genes10090640 (2019). PMC6770913 31450566

[21] Winter S. F. Mitigating Radiotoxicity in the Central Nervous System: Role of Proton Therapy. Curr Treat Options Oncol, doi:10.1007/s11864-023-01131-x (2023). PMC12180537 37728819

[22] Lun M. P., Monuki E. S. & Lehtinen M. K. Development and functions of the choroid plexus-cerebrospinal fluid system. Nat Rev Neurosci 16, 445–457, doi:10.1038/nrn3921 (2015). 26174708 PMC4629451

[23] Lehtinen M. K. The cerebrospinal fluid provides a proliferative niche for neural progenitor cells. Neuron 69, 893–905, doi:10.1016/j.neuron.2011.01.023 (2011). 21382550 PMC3085909

[24] Pellegrini L. Human CNS barrier-forming organoids with cerebrospinal fluid production. Science 369, doi:10.1126/science.aaz5626 (2020). PMC7116154 32527923

[25] Parichha A. Constitutive activation of canonical Wnt signaling disrupts choroid plexus epithelial fate. Nat Commun 13, 633, doi:10.1038/s41467-021-27602-z (2022). 35110543 PMC8810795

[26] Langford M. B. WNT5a Regulates Epithelial Morphogenesis in the Developing Choroid Plexus. Cereb Cortex 30, 3617–3631, doi:10.1093/cercor/bhz330 (2020). 31912879

[27] Bem J. Wnt/beta-catenin signaling in brain development and mental disorders: keeping TCF7L2 in mind. FEBS Lett 593, 1654–1674, doi:10.1002/1873-3468.13502 (2019). 31218672 PMC6772062

[28] Yang A. C. Publisher Correction: Dysregulation of brain and choroid plexus cell types in severe COVID-19. Nature 598, E4, doi:10.1038/s41586-021-04080-3 (2021). 34625744 PMC8500262

[29] Yang A. C. Dysregulation of brain and choroid plexus cell types in severe COVID-19. Nature 595, 565–571, doi:10.1038/s41586-021-03710-0 (2021). 34153974 PMC8400927

[30] Gong J. Three-dimensional in vitro tissue culture models of brain organoids. Exp Neurol 339, 113619, doi:10.1016/j.expneurol.2021.113619 (2021). 33497645

[31] Kompanikova P. & Bryja V. Regulation of choroid plexus development and its functions. Cell Mol Life Sci 79, 304, doi:10.1007/s00018-022-04314-1 (2022). 35589983 PMC9119385

[32] Hou P. S., hAilin D. O., Vogel T. & Hanashima C. Transcription and Beyond: Delineating FOXG1 Function in Cortical Development and Disorders. Front Cell Neurosci 14, 35, doi:10.3389/fncel.2020.00035 (2020). 32158381 PMC7052011

[33] Eulitz J. Increased relative biological effectiveness and periventricular radiosensitivity in proton therapy of glioma patients. Radiother Oncol 178, 109422, doi:10.1016/j.radonc.2022.11.011 (2023). 36435337

[34] Bahn E. Late Contrast Enhancing Brain Lesions in Proton-Treated Patients With Low-Grade Glioma: Clinical Evidence for Increased Periventricular Sensitivity and Variable RBE. Int J Radiat Oncol Biol Phys 107, 571–578, doi:10.1016/j.ijrobp.2020.03.013 (2020). 32234554

[35] Ohashi T., Naganawa S., Iwata S. & Kuno K. Distribution of Gadolinium-based Contrast Agent after Leaking into the Cerebrospinal Fluid: Comparison between the Cerebral Cisterns and the Lateral Ventricles. Magn Reson Med Sci 20, 175–181, doi:10.2463/mrms.mp.2020-0016 (2021). 32641590 PMC8203476

[36] Bozzao A. Cerebrospinal fluid changes after intravenous injection of gadolinium chelate: assessment by FLAIR MR imaging. Eur Radiol 13, 592–597, doi:10.1007/s00330-002-1546-4 (2003). 12594563

[37] Prasongchean W., Vernay B., Asgarian Z., Jannatul N. & Ferretti P. The neural milieu of the developing choroid plexus: neural stem cells, neurons and innervation. Front Neurosci 9, 103, doi:10.3389/fnins.2015.00103 (2015). 25873856 PMC4379892

[38] Sorrells S. F. Human hippocampal neurogenesis drops sharply in children to undetectable levels in adults. Nature 555, 377–381, doi:10.1038/nature25975 (2018). 29513649 PMC6179355

[39] Yang X., Ren H. & Fu J. Treatment of Radiation-Induced Brain Necrosis. Oxid Med Cell Longev 2021, 4793517, doi:10.1155/2021/4793517 (2021). 34976300 PMC8720020

[40] Maharaj A. S. VEGF and TGF-beta are required for the maintenance of the choroid plexus and ependyma. J Exp Med 205, 491–501, doi:10.1084/jem.20072041 (2008). 18268040 PMC2271023

[41] Cheng J. A phase 2 study of thalidomide for the treatment of radiation-induced blood-brain barrier injury. Sci Transl Med 15, eabm6543, doi:10.1126/scitranslmed.abm6543 (2023). 36812346

[42] Meganathan K. Identification of thalidomide-specific transcriptomics and proteomics signatures during differentiation of human embryonic stem cells. PLoS One 7, e44228, doi:10.1371/journal.pone.0044228 (2012). 22952932 PMC3429450

[43] Scotton W. J. Topiramate is more effective than acetazolamide at lowering intracranial pressure. Cephalalgia 39, 209–218, doi:10.1177/0333102418776455 (2019). 29898611 PMC6376637

[44] Thomson J. A. Embryonic stem cell lines derived from human blastocysts. Science 282, 1145–1147, doi:10.1126/science.282.5391.1145 (1998). 9804556

